# ^18^F-FDG-PET/CT for the detection of disease in patients with head and neck cancer treated with radiotherapy

**DOI:** 10.1371/journal.pone.0182350

**Published:** 2017-08-03

**Authors:** Nils Helsen, Dessie Roothans, Bert Van Den Heuvel, Tim Van den Wyngaert, Danielle Van den Weyngaert, Laurens Carp, Sigrid Stroobants

**Affiliations:** 1 Department of Nuclear Medicine, Antwerp University Hospital, Wilrijkstraat 10 Edegem, Belgium; 2 Faculty of Medicine and Health Sciences, University of Antwerp, Universiteitsplein 1, Wilrijk, Belgium; 3 Department of Radiotherapy, ZNA Middelheim, Lindendreef 1 Antwerp, Belgium; University of Chicago, UNITED STATES

## Abstract

**Objective:**

The aim of this study is to evaluate the diagnostic performance of FDG-PET/CT for the detection of residual disease after (chemo)radiotherapy in patients with head and neck squamous cell carcinoma (HNSCC) and to evaluate the prognostic value of the FDG-PET/CT findings.

**Methods:**

Patients with HNSCC who underwent FDG-PET/CT after (chemo)radiotherapy were studied retrospectively.

**Results:**

104 FDG-PET/CT-scans were performed at a median of 13.2 weeks post-treatment (5.4–19.0 weeks). The diagnostic performance was time dependent with decreasing sensitivity and slightly increasing specificity over time. Sensitivity, specificity, PPV and NPV at 9 months after imaging were 91%, 87%, 77% and 95%, respectively. In a logistic regression model, the odds of a correct FDG-PET/CT increased with 33% every additional week after end of therapy (*p* = 0.01) and accuracy plateaued after 11 weeks (97%; *p*<0.001). A complete response on FDG-PET/CT was associated with an overall survival benefit (50.7 versus 10.3 months; *p*<0.001). Residual disease on FDG-PET/CT increased the risk of death 8-fold (*p*<0.001).

**Conclusion:**

FDG-PET/CT is able to detect residual disease after (chemo)radiotherapy, with an optimal time point for scanning between 11–12 weeks after therapy. However, a reevaluation is probably necessary 10–12 months after the FDG-PET/CT to detect late recurrences. In addition, FDG-PET/CT can guide decisions about neck dissection and identifies patients with poor prognosis.

## Introduction

Head and neck cancer is the sixth most common malignancy worldwide [1,2]. About 40% of patients present in an early stage (stage I or II) and are treated with surgery and/or radiotherapy. Combined modality treatment is generally recommended in locoregional disease, with chemoradiation as current standard of care [2–8].

Accurate assessment of treatment response is essential since residual disease in the neck after CRT is present in 30–60% of patients. Even in patients with a clinical complete response, 16–39% have microscopic residual tumor [9–12]. The accuracy of physical examination and conventional imaging is therefore not always sufficient [13]. For this reason, many centers used to perform routine neck dissection after CRT in patients with high risk tumors, even though this procedure is associated with considerable morbidity [10].

Integrated positron emission tomography (PET) with 2-[fluorine-18]-fluoro-2-deoxy-D-glucose (FDG)/ computed tomography (CT) proved to have a high accuracy for detecting residual disease [14,15], suggesting that FDG-PET/CT can be used to defer neck dissection after CRT because of its high negative predictive value (NPV) [14,16,17]. This was recently confirmed in a large randomized trial demonstrating non-inferiority between FDG-PET/CT surveillance and routine neck dissections in N2-N3 head and neck squamous cell carcinoma HNSCC patients [18]. However, further research is required to define the optimal surveillance schedule, including the impact of disease prevalence, the potential differences in detecting early and late recurrences, and the timing of FDG-PET/CT after completing treatment.

## Patients and methods

### Patients

For this retrospective study, all patients who underwent FDG-PET/CT scanning at the Antwerp University Hospital between July 2005 and May 2009, were screened for patients with HNSCC. Eligibility criteria were a histologically confirmed diagnosis of new or recurrent stage I-IVb HNSCC treated with curative intent with radiotherapy with or without chemotherapy and a minimal follow-up time of 12 months or known date of death. Patients with another malignancy five years prior to the diagnosis of HNSCC or patients with distant metastases (stage IVc) were excluded. Due to the retrospective nature of this trial, informed consent and approval of the institutional review board were not required by national law.

### Reference standard

Whenever available, histological examination of a biopsy, tumor resection or neck dissection specimen was used as the reference standard. Otherwise, the follow-up or the time of documented death were used. For the nodal status analysis, confirmation of nodal involvement by other imaging modalities was also accepted since pathological confirmation was not always performed when patients had proven residual disease elsewhere.

### Imaging procedure

All patients underwent FDG-PET/CT between 5–19 weeks after (chemo)radiotherapy for detection of residual disease. FDG-PET/CT was performed in accordance with the guidelines published by the European Association of Nuclear Medicine (EANM) [19]. Patients were instructed to fast for 6 hours prior to their appointment and blood glucose levels were measured prior to the injection of FDG. Combined PET-CT scanning was performed on a Siemens Biograph 6 HIREZ scanner: 60–90 minutes after tracer injection (4 MBq/kg), a whole-body FDG-PET/CT scan was performed (PET acquisition 5 min per bed position, from pelvis to head). PET images were reconstructed iteratively (OSEM; 4 iterations, 8 subsets) with a matrix size of 168x168. A low dose CT-scan (35 mAs, no intravenous contrast) was used for anatomical localization and attenuation correction. Subsequently, every patient received a dedicated head and neck image, with a higher resolution PET (acquisition 10 min per bed position, from vertex to aortic root, reconstructed in a 336x336 matrix; 6 iterations, 16 subsets) and a medium dose CT-scan (85 mAs, without contrast) for attenuation correction and anatomical localization.

### Image interpretation

FDG-PET/CT results were retrieved from the hospital patient record. At the time of reporting the certified nuclear medicine physician was aware of the clinical history and results of other imaging modalities but not of outcome. FDG-PET images were interpreted qualitatively through visual analysis. The reports were retrospectively reviewed and classified into positive, negative or equivocal for residual disease. Reports were called positive when residual focal FDG-uptake was present, of intensity greater than background bloodpool activity or surrounding normal tissue and outside normal anatomic structures seen on CT. On PET/CT, the residual FDG-uptake should fuse to the site of the primary tumor or lymph nodes. If available a comparison was made with the baseline FDG-PET/CT scan to distinguish new lesions. For patients with equivocal reports, images were re-analyzed by an expert nuclear medicine physician and categorized as positive or negative. In case of doubt, the scan was read positive.

### Endpoints and statistical analysis

Study endpoints were to evaluate the diagnostic performance of FDG-PET/CT for the detection of residual disease after CRT, to investigate the impact of end-point censoring, to assess the influence of the time interval between the end of therapy and PET imaging, and determine the prognostic value of the FDG-PET/CT findings. The following test-characteristics were calculated with exact 95% confidence intervals (95% CI): sensitivity, specificity, positive predictive value (PPV), negative predictive value (NPV) and accuracy. Primary analyses were performed at a patient level. In a secondary analysis, we also calculated aforementioned test-characteristics for the nodal status. The effect of end-point censoring was assessed by constructing time-dependent receiver operator characteristics (ROC) curves [20,21]. To study the influence of the timing of FDG-PET/CT after the end of therapy, a binary logistic regression model was used. To define an optimal time point for scanning, a ROC-curve was constructed. The test characteristics of FDG-PET/CT were calculated separately respectively before and after this defined time point and compared using the Pearson chi square or Fisher’s exact test, as appropriate. Due to the time dependency, a standardized interpretation of the reference standard is necessary. For this reason, the overall statistics demonstrate the diagnostic performance 9 months after scanning.

The prognostic value of FDG-PET/CT was evaluated using Kaplan-Meier analysis (with log-rank test) and Cox proportional hazards regression.

IBM SPSS Statistics version 20.0 statistical software was used with alpha set at 5%. Results are reported according to the Standards for the Reporting of Diagnostic accuracy studies (STARD) guideline [22].

## Results

### Patient characteristics

**Fig 1:**
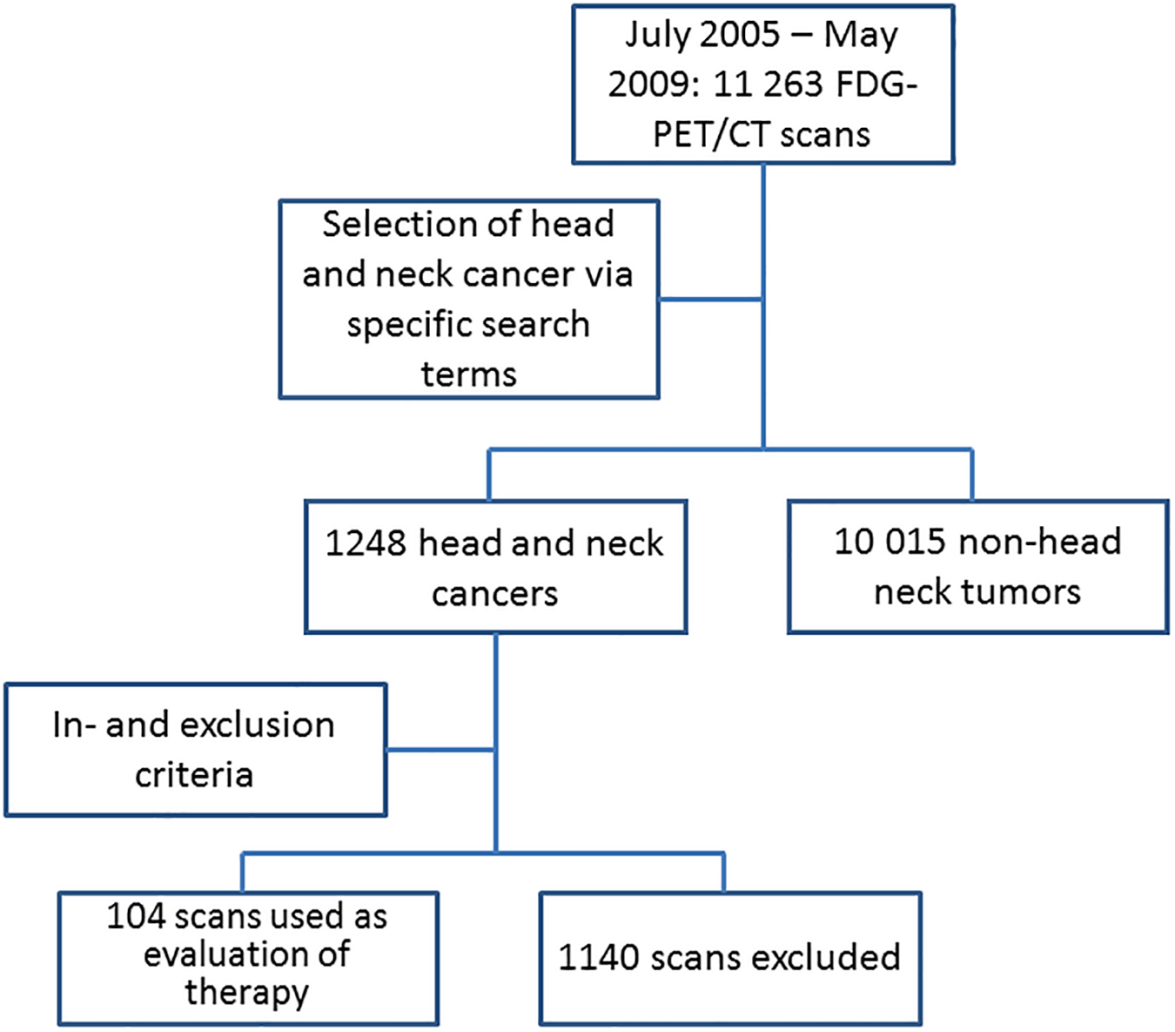
Flow chart illustrating patient selection process.

104 FDG-PET/CT scans in 103 patients were available for analysis (Fig 1). One patient was evaluated twice; the second scan was two years after first line treatment and 2 months after resection and radiotherapy for a recurrence. Table 1 gives an overview of the characteristics of the study population.

**Table 1:** Patient characteristics.

		Study population (n = 103)
Median age (years)		61 (range : 38–87)
Men		83 (80.6%)
Women		20 (19.4%)
Primary tumor	Oropharynx	40 (38.8%)
	Larynx	30 (29.1%)
	Hypopharynx	15 (14.6%)
	Oral cavity	13 (12.6%)
	Nasopharynx	3 (2.9%)
	Unknown	2 (1.9%)
Stage	I	1 (1.0%)
	II	9 (8.7%)
	III	13 (12.5%)
	IVA	71 (68.3%)
	IVB	10 (9.6%)
Therapy	NAC + CRT	40 (38.5%)
	CRT	26 (25.0%)
	S + CRT	15 (14.4%)
	S + RT	9 (8.7%)
	RT	9 (8.7%)
	S + NAC + CRT	3 (2.9%)
	NAC + RT	2 (1.9%)
Median interval after therapy (weeks)		13.2 (range: 5.4–19.0)
Median FU (months)		28.3 (range: 0.4–60.8)

NAC: neoadjuvant chemotherapy; CRT: concommitant chemoradiotherapy; S: surgery; RT: radiotherapy; FU: follow-up

### Diagnostic value of FDG-PET/CT for detection of residual disease

#### Patient level analysis

The actual prevalence of residual disease in our study population at 12 months follow-up was 36% (95% CI 26–46%) (S1 Table). The reference standard was histological examination in 28 (27%) and follow-up in 76 cases (73%).

64/104 FDG-PET/CT scans after the end of (chemo)radiation were scored negative and 39/104 scans positive for residual disease activity. The equivocal scans (7 examinations) were reassessed, 5 examinations were deemed negative after reevaluation and none of them relapsed. One patient was excluded after reassessment due to insufficient follow-up and one scan remained equivocal because of reconstruction artifacts. This scan, with a preference for residual tumor activity at the primary tumor site, was considered positive.

Seven scans were false positive due to moderate FDG-uptake at the primary tumor site or in cervical lymph nodes, which proved to be inflammation. In a patient with sarcoidosis, mediastinal granulomas were wrongly interpreted as distant metastases. In contrast, four scans were false negative with locoregional relapses occurring respectively 4, 6, 7 and 10 months after PET scanning (see also S1 Table)

In one patient, no dedicated head-neck image could be made due to insufficient cooperation. In this patient, one spot of minimal FDG-uptake at the primary tumor site was incorrectly interpreted as inflammatory.

**Fig 2:**
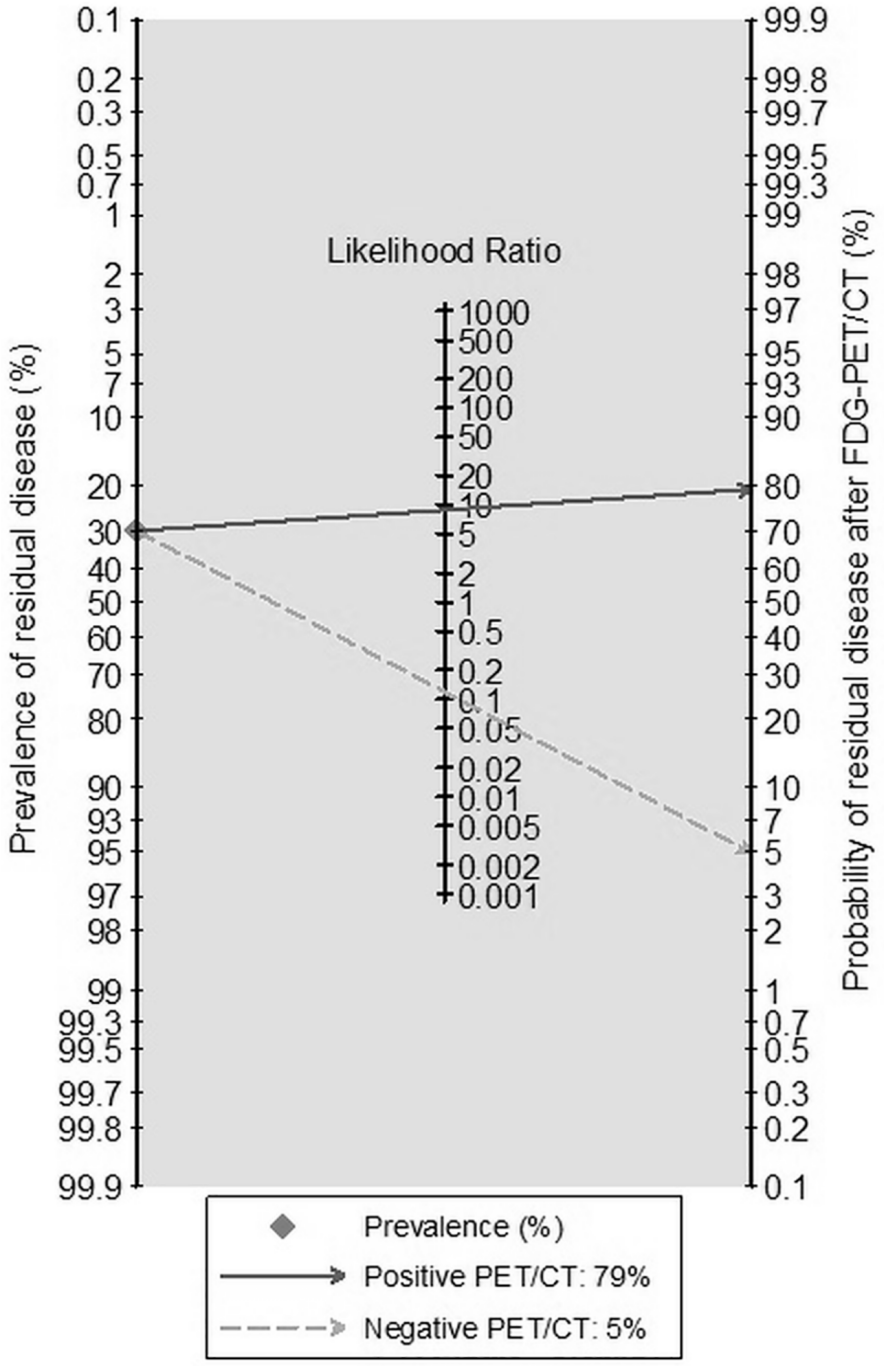
Fagan nomogram illustrating the pre-test probability of residual disease after treatment (prevalence), set at 30%, and the post-test probability after a negative (dotted line) or positive (solid line) FDG-PET/CT scan.

Sensitivity, specificity, PPV and NPV (with 95% confidence intervals) for the detection of residual disease were 91.1% (81.5–100%), 87.0% (79.0–94.9%), 77.3% (64.2–90.4%), and 95.3% (90.0–100%), respectively. In clinical practice, these results imply that for a given pre-test probability of residual disease after treatment of 36% (= prevalence in our study population), a patient with a positive FDG-PET/CT scan will have approximately 80% chance (PPV) of actually having early relapse after treatment. Conversely, negative FDG-PET/CT findings will reduce the probability of residual disease after treatment to only 6% (given an NPV of 94%). The post-test probability of residual disease with a prevalence set at 30% is illustrated in Fig 2.

**Fig 3:**
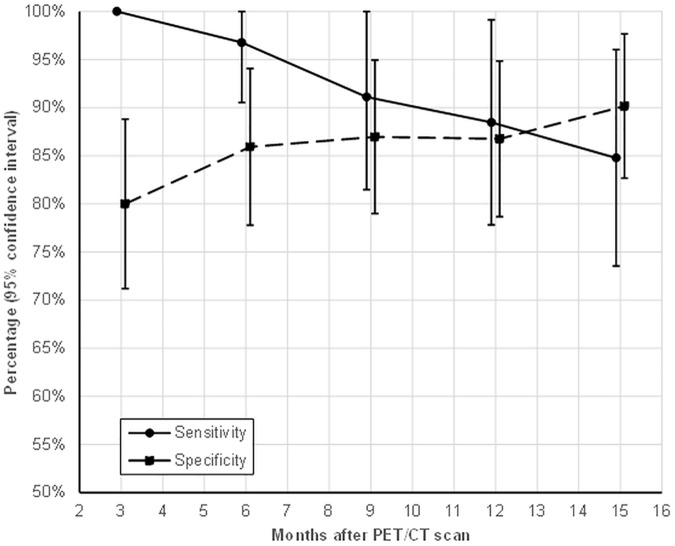
Time dependency of sensitivity and specificity.

To investigate the impact of the time of recurrence on the performance characteristics, an end point censoring analysis was performed (Fig 3). FDG-PET/CT proved to be very powerful to detect recurrences that occur within 9 months after the scan with a sensitivity of 91.1 (95% CI: 81.5–100%). This is in contrast to late recurrences in which the sensitivity gradually decreases to only 84.8% (95% CI: 73.5–96.0%) for recurrences at 15 months after the scan. The specificity on the other hand increases from 80% (71.2–88.8%) 3 months after scanning to 90.2% (82.7–97.7%) at 15 months.

#### Nodal status analysis

Five patients were excluded for this analysis due to the lack of reference standard at the nodal level. The reference standard was histological examination, imaging, unambiguous clinical progression and follow-up in 18 (18%), 7 (7%), 3 (3%) and 71 (72%) cases, respectively. Considering the nodal status only, the sensitivity, specificity, PPV and NPV (with 95% confidence intervals) for the detection of nodal disease were 91% (72–99%), 93% (85–98%), 81% (61–93%), 97% (90–100%). The chance of having locoregional disease is therefore 81% and 3% in case of a positive or negative PET/CT scan respectively, given a pre-test probability of 23%. There were two false negative scans, with an isolated recurrence in the cervical lymph nodes at 19 and 25 months respectively. In one of these two patients, the recurrent primary tumor was correctly identified by PET/CT.

### Influence of timing of FDG-PET/CT after the end of therapy

**Fig 4:**
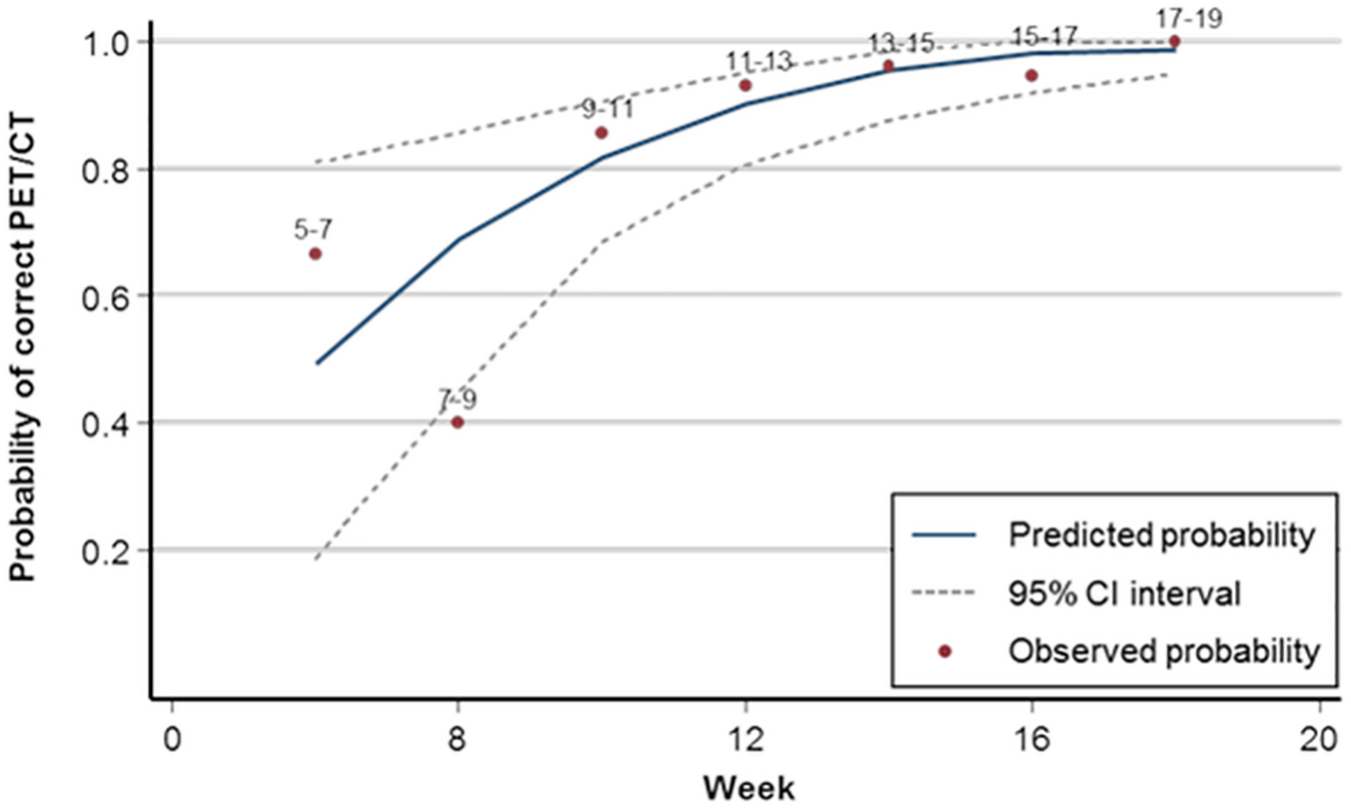
Accuracy depending on the time interval between FDG-PET/CT and the end of therapy. The solid line interval of this model. Real observations from our data (with two week-intervals) are depicted by the dots. The solid line represents the binary logistic regression model. The dotted line represents the 95%-confidence intervals.

The median interval between the end of therapy and FDG-PET/CT was 13.2 weeks (5.4–19.0 weeks). Fig 4 shows the probability of a correct FDG-PET/CT result as a function of the time after the end of therapy, based on a binary logistic regression model. In this model, the odds of a correct FDG-PET/CT result increased by 33% for every additional week after the end of therapy (OR 1.33; 95% CI 1.07–1.66; *p* = 0.01). The probability of a correct result reached a plateau after 11 weeks (Fig 4). Sensitivity and specificity for the detection of residual disease by FDG-PET/CT were calculated separately for scans performed before or after 11 weeks after the end of therapy. Specificity was significantly higher for scans performed at 11 weeks or more after therapy (97% vs 44%; *p*<0.001). Sensitivity was not significantly different (88% vs 91%; *p* = 0.8).

### Prognostic value

In all, 43/103 (42%) patients died, 16 in patients with a negative (16/64, 25%) and 27 patients with a positive (27/40, 68%) PET/CT scan. Seven of sixteen patients with a negative PET/CT scan died because of disease recurrence, 3/16 due to a secondary primary tumor and 6/16 due to non-oncological causes. Most of the 27 patients with a positive FDG-PET/CT scan died due to disease progression. Salvage surgery was performed in 13 patients with positive scan findings.

**Fig 5:**
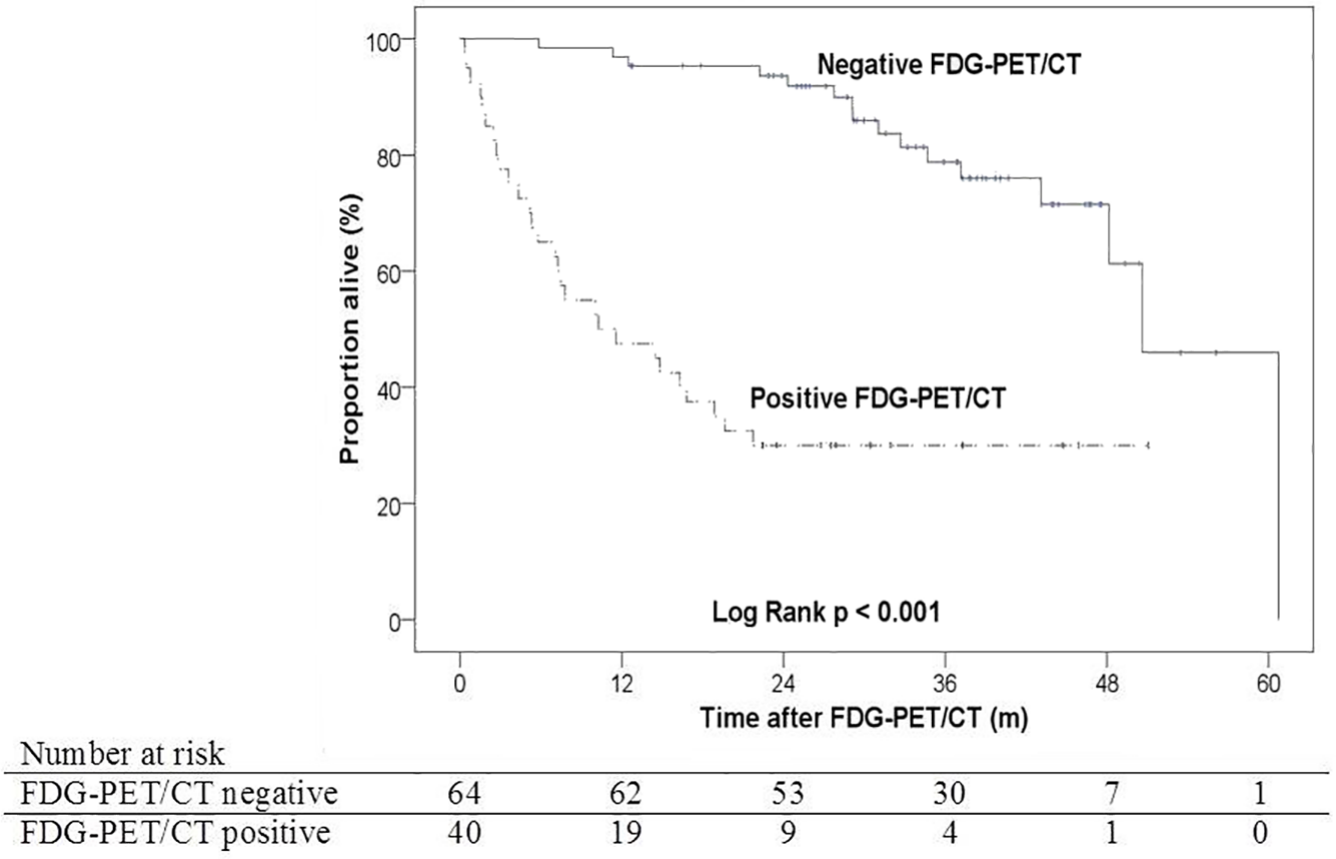
Kaplan-Meier survival curve illustrating the survival of patients with an FDG-PET/CT negative (solid) or positive (dotted) for residual disease. The intersection with the 50% reference line marks the median survival. In a multivariable Cox regression model adjusting for age, sex and tumor stage, a positive FDG-PET/CT was associated with an almost eightfold higher risk of death (HR 7.9; 95% CI 4.0–15.9; p<0.001).

A negative FDG-PET/CT after therapy was associated with a significantly longer median overall survival (50.7 versus 10.3 months; *p*<0.001) (Fig 5). After adjusting for age, sex, and tumor stage, a positive FDG-PET/CT was associated with nearly an eightfold higher risk of death (HR 7.9; 95% CI 4.0–15.9; p<0.001).

A subgroup analysis investigating the impact of the location of the primary tumor and treatment given, was unable to demonstrate any significant differences for the overall and progression free survival. There was also no association between the location of the primary tumor and the ability of FDG-PET/CT to correctly identify the status of the patient.

## Discussion

The debate regarding the need for planned neck dissections in patients with extensive cervical lymph node metastasis (N2 or N3 disease) at initial diagnosis has recently been settled in light of a large randomized trial (564 patients, median follow-up 36 months) demonstrating that FDG-PET/CT surveillance resulted in non-inferior survival (HR 0.92 (95% confidence intervals 0.65–1.32), 2y survival ratio 84,9%) with fewer operations and was more cost effective compared to planned neck dissections (2y survival ratio 81.5%) [18]. The current study confirms the diagnostic value for the detection of residual disease after (chemo)radiotherapy in HNSCC patients in this large study of HNSCC patients receiving integrated FDG-PET/CT.

While the value of FDG-PET/CT in therapy evaluation is supported by other literature data [15–17,23–28], the relationship between FDG-PET/CT accuracy and recurrence timing is unknown. End point censoring analyses found a time-dependent decrease of sensitivity, with lower sensitivity for late recurrences. This is not surprising since late recurrences probably have only microscopic residual disease at the time of imaging with FDG uptake below the detection limit of the PET camera. This finding is potentially of important clinical value in determining the optimal FDG-PET/CT surveillance strategy. Based on these results, a subset of patients may require repeat imaging no longer than 12–15 months after the first FDG-PET/CT scan. Moreover, efforts should be made to optimize the sensitivity of PET acquisition protocols. Rodrigues et al. [29] showed that dedicated head and neck FDG-PET/CT, offered the major advantage of detecting of small lymph node metastases (<15mm), with higher sensitivity (91 vs 70%) and negative NPV (96% vs 89%). Similar results were reported by Yamamoto et al. [30], with a 7.4% higher detection rate of small lymph nodes (5-10mm). This resulted in improved sensitivity for detecting lymph node metastases (83 to 94%), at the cost of a slightly lower specificity (84 vs 81%). In the current study we also performed dedicated head and neck acquisitions but despite this, minimal microscopic disease was not detected.

There also remains considerable debate about the ideal timing of the first FDG-PET/CT after the end of therapy. Many authors state that the accuracy of FDG-PET(/CT) increases with a longer time interval after the end of therapy, with best results between 10 and 12 weeks [14,16], even though as early as 8 weeks has been advocated [15]. A recent retrospective study (n = 247) reported that FDG-PET/CT can be obtained as early as 2 months. However, large differences) in sensitivity (73% vs 93%) between two groups (7–10 vs 11–14 weeks after therapy) were observed [31]. In the first weeks, after radiation, tumor repopulation by viable cells may be insufficient to detect with FDG-PET/CT and, conversely, diffusely increased FDG-uptake due to inflammation can both mask residual disease or be misinterpreted as malignant [24]. From a surgical point, salvage surgery after (chemo)radiation is performed as close as possible to the end of the radiotherapy [32,33], to avoid complications by late fibrotic changes [34]. Therefore, improvements in accuracy of PET/CT with increasing post-therapy intervals need to be balanced with these surgical considerations. Our results suggest an improved accuracy of FDG-PET/CT with time, driven by a significant increase in specificity. The lower bound of this interval was found to be 11 weeks after the end of therapy. Further delaying FDG-PET/CT scanning beyond 11 weeks would increase the risk of surgical complication with little or no additional benefit in diagnostic performance.

Our study also addressed the prognostic role of FDG-PET/CT, demonstrating that prognosis is better in patients with a negative FDG-PET/CT result. In a retrospective study by Passero et al. [28] on 53 patients a higher two year progression free survival was reported in patients with a complete response on FDG-PET/CT examination, namely 92.6 versus 47.9% (*p* = 0.0002). This was in contrast with the results of the survival analysis based on the results of diagnostic CT or clinical examination, which showed no statistically significant association. Castaldi et al. [35] studied the prognostic value of FDG-PET/CT in 26 patients who were scanned 8 to 12 weeks after the end of therapy. The authors noted a significantly higher 2-year disease-free survival in patients with a complete response (73%) versus patients with partial response (50% 2-y DFS) and patients with progression of disease (33%) (*p* = 0.01).

We would also like to highlight some drawbacks of our study. Our study is retrospective in nature, which makes it susceptible to bias, for example selection bias. Although our study population only includes squamous cell carcinoma there was quite some variation in terms of tumor location, tumor stage and treatment. However, we believe that it is a representative sample of the patient population in routine clinical practice. No HPV testing was performed, as this biomarker was not well known in 2005. Like many other centers, we only performed visual image analysis. Although some studies report the use of standard uptake value (SUV) thresholds to discriminate benign from residual disease [36,37], there is no consensus on the optimal threshold. Moreover SUVs are influenced by many factors (reconstructive method, uptake time, injected dose) so translation from SUV thresholds between centers is not straight forward.

## Conclusion

In conclusion, dedicated head neck FDG-PET/CT has excellent diagnostic accuracy to detect residual disease after (chemo)radiotherapy for HNSCC. The optimal time point for the first FDG-PET/CT scan is situated between 11 and 12 weeks after the end of therapy. The inability to detect late recurrences suggests that a reevaluation should be performed 10–12 months after the first scan. However, prospective studies are needed to confirm these findings. FDG-PET/CT has a high prognostic value and can be used to identify patients with a worse prognosis.

## Supporting information

S1 Table All relapsed patients. (DOC)
