# Supplementary material for: 18F-FDG-PET/CT for the detection of disease in patients with head and neck cancer treated with radiotherapy
Source: PLoS One. 2017 Aug 3;12(8):e0182350. doi: 10.1371/journal.pone.0182350 (PMC5542639; doi:10.1371/journal.pone.0182350)
Supplement: S1 Table — (DOC) [file pone.0182350.s001.doc]

| **Table S1: All relapsed patients** | | |  |  |  |  |  |  |  |
| --- | --- | --- | --- | --- | --- | --- | --- | --- | --- |
| **SeqID** | **Location** | **Baseline cTNM** | **Treatment** | **FDG-PET/CT** | | **Relapse** | | | **Deceased** |
|  |  |  |  | # weeks after therapy | PET finding | Time after scanning (months) | Location | Treatment |  |
| 1 | Oropharynx | T4N3M0 | NAC + CRT | 15 | + | 0 | PL + LN + M | CH | Yes |
| 2 | Oropharynx | T3N2cM0 | NAC + CRT | 13 | + | 5 | PL + LN + M | CH | Yes |
| 3 | Oropharynx | T3N2bM0 | NAC + CRT | 13 | + | 3 | LN + M | CH | Yes |
| 4 | Oropharynx | T3N2bM0 | NAC + CRT | 11 | + | 7 | LN + M | NA | Yes |
| 5 | Oropharynx | T2N1M0 | NAC + CRT | 17 | + | 1 | PL + M | CH | Yes |
| 6 | Oropharynx | T4N2cM0 | NAC + CRT | 15 | + | 0 | PL + LN | CH | Yes |
| 7 | Oropharynx | T2N2aM0 | NAC + RT | 16 | + | 1 | PL | CRT | Yes |
| 8 | Oropharynx | T4N2M0 | CRT | 11 | + | 0 | PL | CH | Yes |
| 9 | Oropharynx | T4N2aM0 | CRT | 11 | + | 1 | PL + LN | S | Yes |
| 10 | Oropharynx | rT4N3M0 | S + CRT | 15 | + | 3 | PL | CH | Yes |
| 11 | Oropharynx | T3N2bM0 | S + CRT | 12 | + | 2 | PL + LN + M | CH | Yes |
| 12 | Oropharynx | T1N0M0 | S + CRT | 13 | + | 5 | LN | S | No |
| 13 | Oropharynx | T2N0M0 | S + CRT | 15 | + | 1 | M | CH | Yes |
| 14 | Oral Cavity | T3N2cM0 | NAC + CRT | 13 | + | 1 | LN | S | No |
| 15 | Oral Cavity | T4N3M0 | S + RT | 10 | + | 3 | LN + M | CH | Yes |
| 16 | Oral Cavity | T2N1M0 | S + RT | 13 | + | 6 | LN + M | RT | Yes |
| 17 | Oral Cavity | T3N0M0 | RT | 12 | + | 1 | PL | S | Yes |
| 18 | Nasopharynx | T2N3M0 | CRT | 10 | + | 1 | PL | CH | Yes |
| 19 | Larynx | T4N2cM0 | CRT | 9 | + | 1 | LN + M | CH | Yes |
| 20 | Larynx | T2N2bM0 | CRT | 9 | + | 1 | LN | S | No |
| 21 | Larynx | T1N2cM0 | CRT | 13 | + | 1 | LN | S | No |
| 22 | Larynx | T4N2cM0 | S + NAC + CRT | 16 | + | 0 | LN + M | CH | Yes |
| 23 | Larynx | T2N0M0 | S + CRT | 10 | + | 5 | PL + LN | S | Yes |
| 24 | Larynx | T4N1M0 | S + CRT | 9 | + | 0 | LN | CH | Yes |
| 25 | Larynx | T4N0M0 | S + RT | 6 | + | 0 | LN | NA | Yes |
| 26 | Larynx | T4aN1M0 | S + RT | 19 | + | 1 | PL + LN + M | CH | Yes |
| 27 | Hypofarynx | T2N3M0 | NAC + CRT | 12 | + | 1 | LN | S | No |
| 28 | Hypofarynx | T3N2bM0 | NAC + CRT | 16 | + | 4 | M | nvt | No |
| 29 | Hypofarynx | T4N0M0 | NAC + CRT | 10 | + | 3 | PL | CH | Yes |
| 30 | Hypofarynx | T3N2bM0 | NAC + CRT | 15 | + | 2 | LN | S | Yes |
| 31 | Hypofarynx | T4N3M0 | NAC + CRT | 13 | + | 3 | PL + LN | CH | Yes |
| 32 | Hypofarynx | T4aN2bM0 | NAC + CRT | 6 | + | 1 | LN | S | No |
| 33 | Oropharynx | T2N2aM0 | S + CRT | 16 | EQ => + | 3 | PL + M | CH + S | Yes |
| 34 | Oral Cavity | T4N0M0 | S + RT | 8 | - | 4 | PL | S | Yes |
| 35 | Larynx | T4N2cM0 | NAC + CRT | 13 | - | 7 | OTH | CH | Yes |
| 36 | Larynx | T1N1M0 | RT | 12 | - | 6 | PL | S | Yes |
| 37 | Hypofarynx | T4N2cM0 | NAC + CRT | 13 | - | 10 | PL +LN | S | Yes |
| NAC: neoadjuvant chemotherapy; CRT: concommitant chemoradiotherapy; S: surgery; RT: radiotherapy, CH: chemotherapyn NA: not applicable, PL: primary lesion, LN: lymph node, M: metasasis, EQ: equicoval | | | | | | | | | |
|  | | | | | | | | | |
